# Supplementary material for: Emergence and disruption of cooperativity in a denitrifying microbial community
Source: ISME J. 2025 May 11;19(1):wraf093. doi: 10.1093/ismejo/wraf093 (PMC12146267; doi:10.1093/ismejo/wraf093)
Supplement: Supplemental_Figures_warf093 [file supplemental_figures_warf093.pdf]

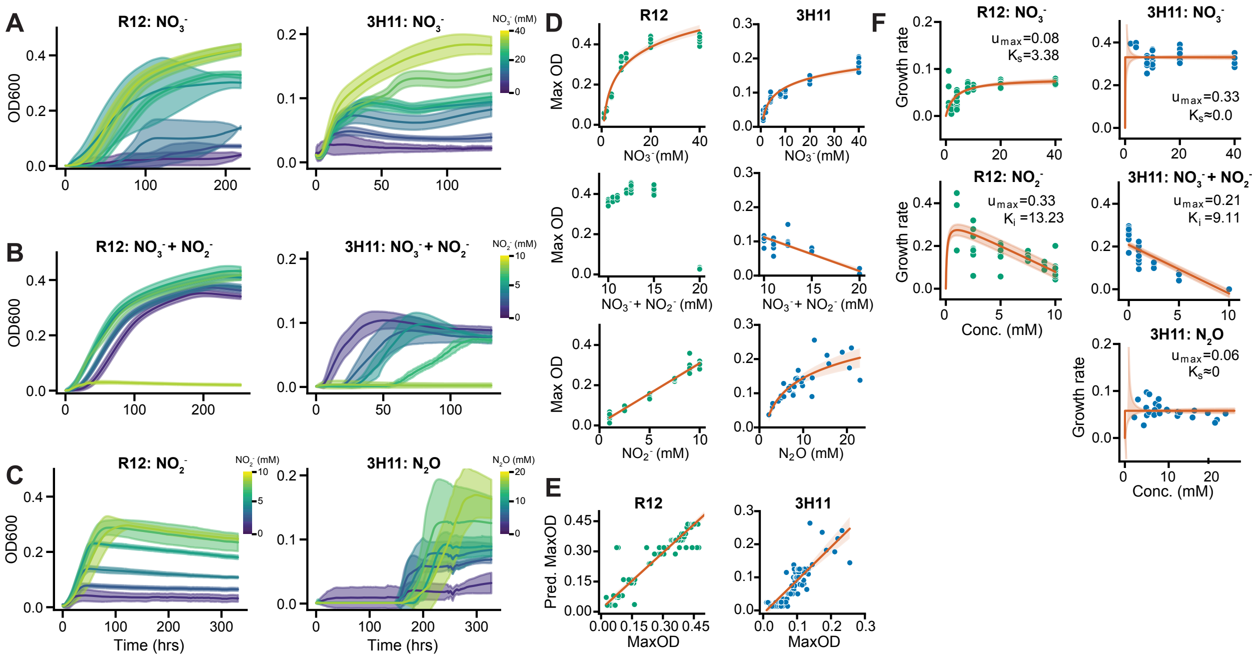

Supplemental figure 1. Growth characteristics of 3H11 and R12 across variation in substrate concentration. (**A**) Growth of R12 and 3H11 across variation in initial NO_3^- concentration. Trendlines display average trajectories across eight replicates and error bands display standard deviations. (**B**) Growth of R12 and 3H11 across variation in initial NO_2^- concentration supplemented with 10 mM NO_3. (**C**) Growth of R12 and 3H11 across variation in initial NO_2^- and N_2O concentration respectively. Trendlines display average growth trajectories across eight replicates and error bands display standard deviations. Trendlines are colored by respective substrate concentrations. (**D**) Maximum OD600 achieved by 3H11 and R12 across growth conditions as a function of substrate concentration. OLS linear or logarithmic fits to data are displayed with 95% confidence intervals where appropriate. (**E**) Predicted maximum OD600 vs measured maximum OD600 using multi-linear fit to linear portions of substrate vs maximum OD600 data. (**F**) Maximum growth rate as a function of substrate concentration across conditions for 3H11 and R12. Fits of Monod substrate kinetics or Monod product inhibition kinetics displayed with 95% confidence intervals and associated parameters.

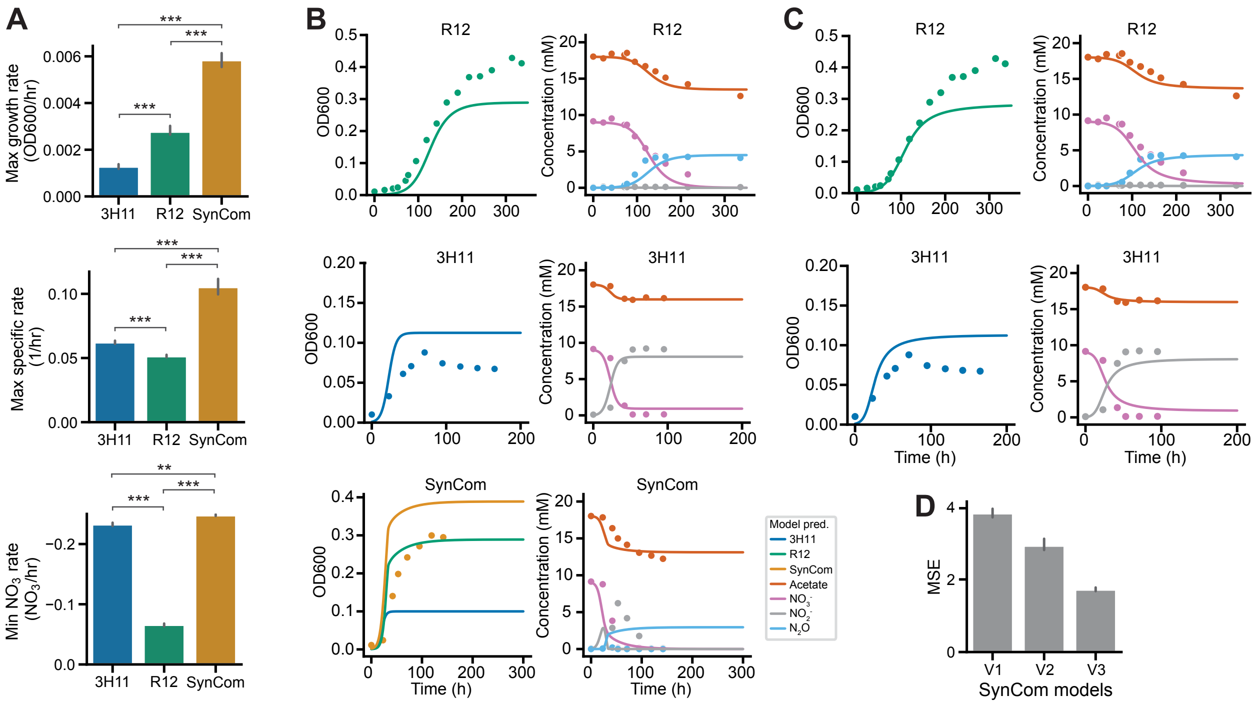

Supplemental figure 2. Kinetic modeling of individual monocultures and SynCom growth characteristics. (**A**) Comparison of monoculture and SynCom maximum growth rates, maximum specific rates and minimum NO_3^- rate from the transcriptomics growth data. (**B**) Predicted growth dynamics and associated data for initial kinetic model formulations for R12, 3H11 and the SynCom. (**C**) Updated growth dynamics predictions with models which included NO_3^- inhibition. Model predictions are represented using solid lines, data are averages across replicates and are represented using circular points. SynCom models assume NO_2^- and N_2O are exchanged. (**D**) Comparison of mean standard error (MSE) for three iterations of SynCom kinetic models. MSE computed across both growth and metabolite data. Bars indicate comparisons for which differences were significant using Welch's t-test. *, $P < 0.05$; **, $P < 0.01$; ***, $P < 0.001$.

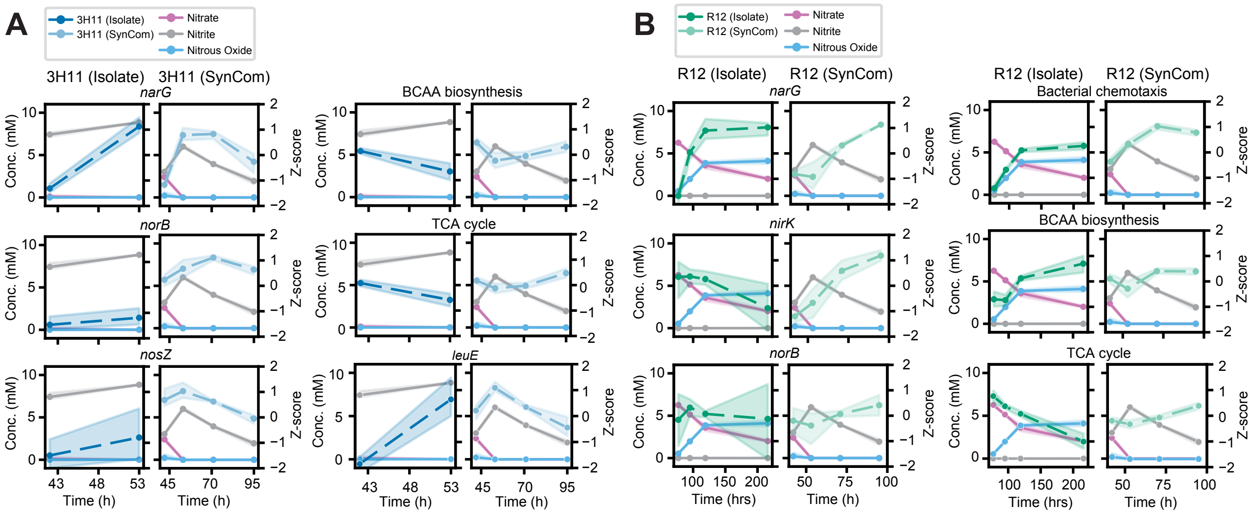

Supplemental figure 3. Time resolved expression dynamics of denitrification genes and select pathways. (**A**) 3H11 Expression dynamics for specific genes and pathways. (**B**) R12 Expression dynamics for specific genes and pathways. Points and trend lines represent averages of replicate samples in the case of genes and averages across genes and samples in the case of pathways. Error bands display standard deviations.

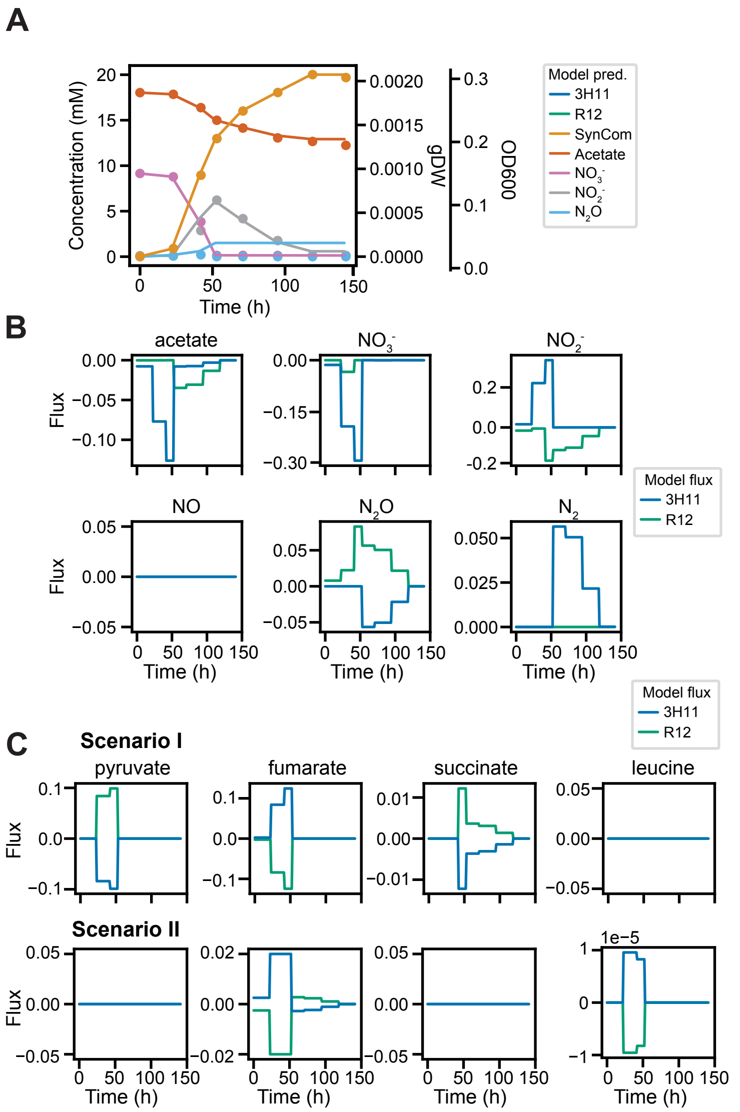

Supplemental figure 4. SynCom genome scale metabolic model predicts exchange of TCA cycle intermediates and leucine. (**A**) Genome scale metabolic model growth predictions and associated growth data used to constrain metabolite exchange fluxes. Acetate, NO_3^-, and NO_2^- consumption and production rates, calculated for each pair of time points, were used to constraints model predictions. Model predictions are displayed using solid lines and represent cumulative changes in biomass and concentrations resulting from fluxes at each time step. Data are averages across samples and are represented using circular points. (**B**) Representative predictions for acetate, NO_3^-, NO_2^-, NO, N_2O, and N_2 across all time steps. Contributions to total SynCom flux from 3H11 (blue) and R12 (green) are displayed. (**C**) Two possible exchange

scenarios predicted by the model are displayed. In scenario I 3H11 provides fumarate and receives both pyruvate and succinate, this is a model default. In scenario II, pyruvate and succinate are blocked which facilitates the exchange of leucine.

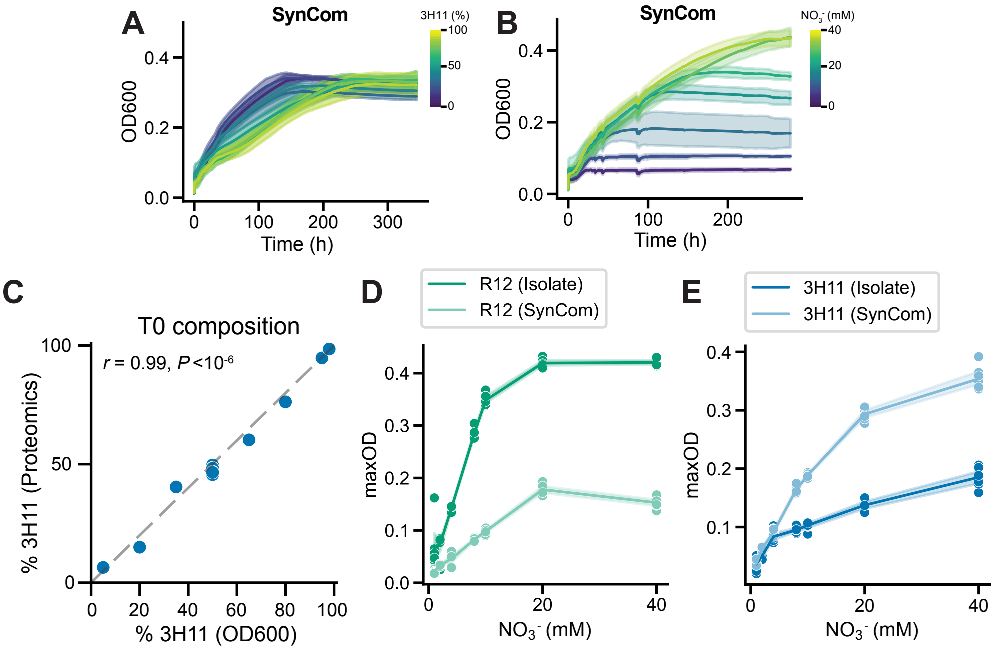

Supplemental figure 5. SynCom growth characteristics across variation in initial community composition and NO_3^- concentration. (**A**) Growth of the SynCpm across variation in initial community composition (3H11 proportion). Trendlines display average trajectories across eight replicates and error bands display standard deviations. Trendlines are colored by 3H11 proportion. (**B**) Growth of the SynCpm across variation in initial NO_3^- concentrations. Trendlines display average trajectories across eight replicates and error bands display standard deviations. Trendlines are colored by NO_3^- concentration. (**C**) Correlation between inoculum 3H11 proportion estimated by OD600 and proteomics. Dashed line indicates a 1:1 relationship. (**D**) Comparison of maximum OD600 achieved by R12 in monoculture vs R12 in the SynCom as a function of NO_3^- concentration. (**E**) Comparison of maximum OD600 achieved by 3H11 in monoculture vs 3H11 in the SynCom as a function of NO_3^- concentration.
